# Novel Polymer Gels: Synthesis, Properties, and Applications

**DOI:** 10.3390/gels11080598

**Published:** 2025-08-01

**Authors:** Amin Babaei-Ghazvini

**Affiliations:** Department of Chemical and Biological Engineering, University of Saskatchewan, 57 Campus Drive, Saskatoon, SK S7N 5A9, Canada; amin.babaei@usask.ca; Tel.: +1-306-966-6498

## 1. Introduction

Polymer gels are a versatile class of soft, semi-solid materials characterized by a three-dimensional cross-linked network that can absorb significant amounts of solvent [1]. In recent years, they have attracted tremendous interest due to their exceptional properties—including flexibility, tunable viscoelasticity, stimuli-responsiveness, biocompatibility, and biodegradability—which allow them to function in unique ways not possible with traditional rigid materials [2,3,4]. As a result, polymer gels find use across a wide range of fields, appearing in everyday products (foods, cosmetics, adhesives) as well as in high-tech applications such as biomedical devices, sensors, and environmental technologies [5,6,7]. Their ability to transition between liquid-like and solid-like behavior and to respond to external stimuli (e.g., pH, temperature, light) makes them indispensable for creating smart and adaptive systems.

Cutting-edge research on polymer gels is rapidly advancing along multiple frontiers. **Synthesis methods** are evolving beyond conventional approaches: new chemical and physical crosslinking techniques (including enzymatic routes and click chemistry) are being developed to precisely control network architecture, while *green* synthesis processes emphasize sustainability by avoiding toxic reagents and harsh conditions [8]. Simultaneously, researchers are intensively studying **properties** of polymer gels to tailor their performance. Key focus areas include understanding the relationships between gel structure and rheological behavior (viscosity, viscoelastic moduli, and flow characteristics under stress), enhancing stimuli-responsiveness (e.g., gels that swell, shrink, or change stiffness in response to pH, temperature, or electric fields), and imparting self-healing capabilities that allow gels to recover after mechanical damage [9]. These efforts aim to design gels that can maintain stability and function under complex conditions while offering dynamic, responsive behavior.

The **applications** of polymer gels are extraordinarily diverse, highlighting their interdisciplinary importance. In the biomedical realm, hydrogels serve as drug delivery vehicles (capable of controlled release and targeted delivery) and as scaffolds for tissue engineering and wound healing. In robotics and electronics, flexible gel-based sensors and actuators can mimic soft tissues or respond to environmental cues, enabling innovations in wearable devices and soft robots. Environmental and energy applications have also emerged, with gels being used for water purification, oil spill cleanup, and as functional components in energy storage systems [10,11]. Even in traditional industries, such as construction and oil recovery, polymer gels contribute to novel solutions—for example, as binders in sustainable building materials or as fluid loss control agents in drilling. Given this broad scope, polymer gels serve as a **bridge** between chemistry, materials science, biology, and engineering, offering customizable solutions to complex problems across sectors.

Considering these developments, the *Gels* journal launched this Special Issue, “Novel Polymer Gels: Synthesis, Properties, and Applications”, to capture the latest advances and foster knowledge exchange in this vibrant field. This Special Issue comprises seven contributions (four original research articles and three review articles) that collectively showcase the versatility and innovation in contemporary polymer gel research. These papers span multiple disciplines and application domains—from petrochemical engineering to biomedicine and from environmental science to construction engineering—reflecting the truly interdisciplinary nature of polymer gel science. In the following section, we provide an overview of each contribution, highlighting the study’s aims, key findings, and relevance to the broader context of gel materials and technology.

## 2. Overview of the Papers Published in This Special Issue

The paper “**Study on Filter Cake Removal Fluid of EZFLOW Weak Gel Drilling Fluid**” by Hu et al. investigates an innovative application of polymer gels in oilfield drilling operations. The authors examine the unique internal architecture and rheological behavior of *EZFLOW*, a weak gel-based drilling fluid widely used in horizontal wells, which forms a distinctive reversible network structure that endows it with exceptional viscosity and flow properties. While this gelled drilling fluid effectively plugs micro-pores and fractures in the rock formation (through deformable polymer aggregates and particulate bridging agents), it also leaves behind a dense *filter cake* that can impair well productivity. To address this, Hu et al. developed a specialized filter cake removal fluid consisting of a retarded acidic solution (to degrade the polymer gel and dissolve the temporary plugging particles) and a corrosion inhibitor (to protect wellbore integrity). Laboratory tests demonstrated that this removal fluid completely dissolves the filter cake, achieving over 95% permeability recovery in core samples. The study’s findings are highly relevant for the petroleum industry, showing how polymer gel chemistry can be leveraged to improve drilling fluid performance and subsequently mitigate formation damage in an environmentally compatible way.

The second article, “**Hydration and Hardening Properties of High Fly-Ash Content Gel Material for Cemented Paste Backfill Utilization**” by Xiao et al., explores the use of polymer gel technology to create a sustainable cementing material for mining applications. The authors propose a novel cemented paste backfill (CPB) binder in which **40%** of ordinary Portland cement is replaced with a low-quality Class F fly ash—an industrial by-product—to reduce cost and carbon footprint in underground mine backfilling. They conducted comprehensive experiments (including X-ray diffraction, mercury porosimetry, compressive strength tests, and thermogravimetric analysis) on this fly ash-based gel binder, termed FCM, using three different mine tailings to assess its performance. The results show that the FCM binder can achieve ~72% of the 28-day compressive strength of a traditional cement binder while costing less than half as much and requiring no energy-intensive clinker production. Microstructural analysis revealed that the hydration of FCM proceeds through five stages, yielding ettringite and other gel-like hydration products; by 28 days, the FCM-hardened paste exhibits a slightly higher porosity than OPC paste but with a refined pore structure and sufficient strength for mine support requirements. This work demonstrates an important trend in construction materials: **polymer-modified and waste-derived gels** can replace a significant portion of cement, providing economic and environmental benefits without drastically compromising performance. The study’s implications extend to greener mining practices and the development of low-CO_2_ building materials.

The third paper, “**A Novel Biomineralized Collagen Liquid Crystal Hydrogel Possessing Bone-like Nanostructures by Complete In Vitro Fabrication**” by Li et al., delves into the biomimetic synthesis of polymer gels for regenerative medicine. Inspired by natural bone, which has an intricate nanoscale composite structure of collagen fibrils mineralized with hydroxyapatite, the authors set out to fabricate a **bone-mimicking hydrogel scaffold** entirely in vitro. They combined two advanced techniques: (1) the creation of a *collagen liquid crystal hydrogel (CLCH)*—a highly ordered, dense collagen network formed by self-assembly of collagen macromolecules at elevated pH—and (2) the *polymer-induced liquid precursor (PILP)* process for mineralization. Using this approach, Li et al. successfully induced intrafibrillar mineralization within the collagen hydrogel by first infiltrating it with amorphous CaCO_3_ via the PILP method and then chemically transforming the CaCO_3_ into nano-hydroxyapatite crystals in situ. The resulting composite hydrogel reproduced both the **organic matrix orientation** and the **inorganic mineral dispersion** characteristic of natural bone at the nanoscale. This entirely laboratory-based fabrication of bone-like material is a significant innovation, as it circumvents the need for using actual bone tissue or cells. The study provides a proof-of-concept for designing next-generation biomaterials—particularly for bone tissue engineering—where the **structural hierarchy and composition of native bone** are closely emulated to achieve improved integration and mechanical performance in clinical applications.

In “Enhancing Load-Bearing Capacity of Calcareous Sands through Gel Stabilization: A Mechanical and Material Characterization Study”, Gu et al. address a geotechnical engineering challenge using polymer gel stabilization. Calcareous sands (composed largely of calcium carbonate from marine biogenic sources) are known to have high intragranular porosity, fragile grains, and generally poor cementation, which together result in low strength unsuitable for supporting heavy structures. This study investigated gel-based stabilization as a method to improve the mechanical properties of such sands, blending a polymeric gel binder with the sand at various concentrations (5–22% by weight) and curing the mixtures for periods between 3 and 28 days. Two types of binders were considered (a cement-based gel and a gypsum-based gel), and the treated samples were subjected to unconfined compressive strength tests and triaxial shear tests to evaluate improvements in load-bearing capacity. The experiments showed that introducing gel binders significantly increases the strength and stiffness of calcareous sand: gel-stabilized samples exhibited higher cohesion and secant elastic moduli, and their stress–strain curves demonstrated pronounced strain hardening and dilation behavior (as opposed to the weak, contractive behavior of untreated sand). Moreover, longer curing times and higher gel contents led to better mechanical performance, indicating that the gel forms a reinforcing matrix that continues to develop over time. By establishing a positive correlation between gel stabilization and enhanced geotechnical properties, this work highlights a novel use of polymer gels in civil engineering—one that could enable construction on calcareous sand deposits (e.g., in coastal or desert environments) that would otherwise require costly ground improvement. It underscores the cross-disciplinary innovation where insights from polymer materials are applied to solve problems in soil mechanics.

This Special Issue also features three comprehensive review articles that synthesize recent progress in specific subfields of polymer gels. The first review, “**Biomaterials in Postoperative Adhesion Barriers and Uterine Tissue Engineering**” by Fazel Anvari-Yazdi et al., provides an extensive overview of gel-like biomaterials used to prevent postoperative adhesions—fibrous internal scars that often form after surgeries, causing complications such as chronic pain or infertility. This article surveys a broad spectrum of material strategies for anti-adhesion barriers with a focus on **biopolymer gels** and related scaffolds that can be applied to the uterine or abdominal tissues. The authors discuss natural polymeric materials (including collagen and gelatin hydrogels, fibrin gels, silk fibroin, and polysaccharide-based gels, such as those from alginate, chitosan, or cellulose derivatives), evaluating how their biodegradation rates, mechanical integrity, and biocompatibility affect performance in preventing tissue adhesions. They also review synthetic polymers (such as PEG, PLGA, and PCL) that have been engineered into films or hydrogel coatings, noting that these allow tunable degradation (from days to months) and can be loaded with anti-inflammatory or anti-fibrotic drugs. Notably, Fazel Anvari-Yazdi et al. highlight **emerging design innovations**—for example, multi-layered barrier gels created by electrospinning or 3D bioprinting—which show improved efficacy in preclinical models by better mimicking tissue structure and controlling drug release. Despite these advances, the review points out persisting challenges such as the need for more comparative data on material performance and clearer definitions of ideal mechanical properties for adhesion barriers. By consolidating current knowledge and identifying gaps, this paper guides future development of polymer gel-based solutions in postoperative care and uterine tissue engineering, underlining the importance of interdisciplinary collaboration between materials scientists and clinicians.

The second review, “**Mechanical Properties of Cement-Based Gel Composites Reinforced by Plant Fiber: A Review**” by Zhang et al., examines how natural fibers can be incorporated into cementitious gels to create more sustainable and ductile construction materials. Plant fiber-reinforced cement-based composites (often called *PFRCCs*) are essentially concrete or mortar in which a polymer-like gel matrix of cement hydrates is interpenetrated by lignocellulosic fibers (e.g., from hemp, sisal, or bamboo). This review first summarizes the **material characteristics of plant fibers** relevant to cement reinforcement—including their chemical composition, mechanical properties, and susceptibility to degradation in alkaline cement pore solution. It then discusses various **fiber and matrix modification techniques** (such as fiber surface treatments, use of supplementary cementitious materials, or polymer admixtures) that improve fiber–matrix bonding and durability. Zhang et al. go on to collate findings from numerous studies on the static and dynamic mechanical behavior of PFRCCs: a clear trend is that adding plant fibers enhances the **toughness**, flexural strength, and tensile capacity of cement-based gels, often at the expense of a slight reduction or variability in compressive strength. The fibers act as bridges across cracks, increasing energy absorption and ductility, which is reflected in higher post-cracking resistance and improved fracture behavior of the composites. The review also summarizes predictive models for composite mechanical properties and examines the **interfacial transition zone** between fibers and the cement gel matrix, noting that this interface is crucial for load transfer and can be improved by proper fiber treatment to resist the alkaline environment. Overall, the authors conclude that plant fiber-reinforced gel composites are promising “*green*” building materials with broad application prospects, from reducing carbon emissions (by partially replacing cement) to imparting desirable mechanical characteristics in buildings and infrastructure. By providing this cohesive overview, the review by Zhang et al. aims to accelerate the adoption of sustainable fibrous gels in construction and encourages further research to optimize their long-term performance.

The third review paper, “**Microbial Mineral Gel Network for Enhancing the Performance of Recycled Concrete: A Review**” by Zheng et al., explores the innovative use of microbial-induced calcium carbonate precipitation (MICP) to form in situ **mineral gel networks** within recycled concrete (RC). This bio-mediated process utilizes the metabolic activity of specific microorganisms to deposit calcium carbonate gels in the pores of recycled aggregates, thereby improving inherent weaknesses such as high porosity and poor interfacial bonding. The authors discuss how this mineral gel network enhances concrete strength by 5–30% and offers superior environmental sustainability compared to conventional RC reinforcement techniques. The review covers the biochemical pathways of MICP, types of microorganisms used, critical parameters influencing gel formation, and strategies to optimize MICP for mechanical property enhancement. Furthermore, it assesses emerging prospects such as embedding mineralizing spores for **self-healing concrete** applications. By integrating microbiology, materials engineering, and sustainability, this paper highlights a promising direction for next-generation **eco-friendly construction materials** based on microbial gels.

## 3. Conclusions and Outlook

The research and reviews presented in this Special Issue underscore the **remarkable versatility and interdisciplinary nature** of modern polymer gel science. Several key themes emerge from these six contributions. First, *innovation in synthesis and design* is enabling gels to meet specialized needs: from **environmentally friendly gel formulations** (e.g., low-CO_2_ cementitious binders and biodegradable drilling fluids) to **biomimetic architectures** (bone-like collagenous gels) and **functional hybrids** (fiber-reinforced structural gels). These advances demonstrate how tailoring the molecular structure or composite makeup of gels can translate into tangible improvements in performance across vastly different contexts—improving oil recovery in energy engineering, enhancing the sustainability of construction materials, or creating medically safe interfaces in the human body.

Second, the studies highlight the importance of understanding and optimizing **gel properties** (mechanical, rheological, and interfacial). Whether it is the rheology of a drilling gel that must balance viscosity and flowback or the mechanical stiffness of a tissue-engineered hydrogel that must emulate natural tissue, the ability to characterize and tune gel behavior is crucial. There is a trend toward gels that are *multi-functional*: for instance, materials that combine high mechanical strength with self-healing or stimuli-responsive capabilities, or biomedical gels that provide physical barrier functions while delivering drugs or bioactive cues. The interdisciplinary insights from this Special Issue—drawing on polymer chemistry, materials engineering, nanotechnology, biotechnology, and more—illustrate how complex property requirements can be met by clever gel design and formulation.

Third, the contributions emphasize **sustainability and translational potential**. Using industrial by-products (fly ash, plant fibers) in polymer gel matrices speaks to a larger movement in materials science toward circular economy principles and reducing the carbon footprint of materials. Several works also address scalability and real-world implementation: for example, developing a filter cake removal fluid that can be deployed in actual drilling operations or improving sand stabilization techniques applicable to construction projects. The review articles further identify practical challenges and gaps (such as long-term durability of fibrous cement gels or standardizing performance criteria for adhesion barriers) that must be addressed to move these innovations from the lab to the field or clinic. Addressing these challenges will likely be a focus of future research.

Looking ahead, the field of polymer gels is poised for exciting growth. **Future research** will likely delve deeper into *smart gel systems*—materials that not only serve a single function but can sense, adapt, and interact with their environment in real time. Examples include self-healing infrastructure gels that repair cracks autonomously, stimuli-responsive drug delivery gels that release therapeutics on demand, or gels that serve as 4D-printed components changing shape over time. Additionally, improving the **biocompatibility and bio-functionality** of gels will remain critical for biomedical uses, as will ensuring the **environmental safety** of gels used in large-scale applications (such as those in water treatment or soil stabilization). Collaboration across disciplines will be essential; chemists, engineers, biologists, and clinicians must work together to harness the full potential of polymer gels.

In conclusion, the studies in this Special Issue illustrate that polymer gel research is not only thriving but also branching out in diverse directions with significant societal impact. By combining fundamental scientific advances with an eye toward practical applications, these works collectively push the frontier of what polymer gels can achieve. We anticipate that the novel concepts and findings reported here will inspire further developments, drive new collaborations, and ultimately lead to **next-generation polymer gel technologies** that are smarter, greener, and more impactful than ever before.
